# Topographical Distribution and Phenotype of Resident Meibomian Gland Orifice Immune Cells (MOICs) in Mice and the Effects of Topical Benzalkonium Chloride (BAK)

**DOI:** 10.3390/ijms23179589

**Published:** 2022-08-24

**Authors:** Ching Yi Wu, Mengliang Wu, Xin Huang, Ben J. Gu, Carole Maldonado-Codina, Philip B. Morgan, Laura E. Downie, Holly R. Chinnery

**Affiliations:** 1 Department of Optometry and Vision Sciences, Faculty of Medicine Dentistry and Health Sciences, The University of Melbourne, Parkville, VIC 3010, Australia; 2 Division of Pharmacy and Optometry, The University of Manchester, Oxford Road, Manchester M13 9PR, UK; 3 The Florey Institute, The University of Melbourne, 30 Royal Parade, Parkville, VIC 3052, Australia

**Keywords:** meibomian gland, resident immune cell, dendritic cell, benzalkonium chloride

## Abstract

Meibomian gland orifices (MGOs) are located along the eyelid margin and secrete meibum into the tear film. The profile of resident innate immune cells (ICs) at this site is not well understood. The distribution and phenotype of resident ICs around MGOs in mice was investigated and herein defined as MGO-associated immune cells (MOICs). The effect of topical 0.1% benzalkonium chloride (BAK) on MOICs was also assessed. Eyelids from healthy CD11c^eYFP^ and Cx3cr1^gfp/gfp^ mice aged three or seven months were compared. ICs were identified as CD11c^+^, Cx3cr1^+^, and MHC-II^+^ using four-colour immunostaining and confocal microscopy. MOIC density was variable but clustered around MGOs. There were more CD11c^+^ MOICs in three-month-old compared with seven-month-old mice (three-month-old: 893 ± 449 cells/mm^2^ vs. seven-month-old: 593 ± 493 cells/mm^2^, *p* = 0.004). Along the eyelid margin, there was a decreasing gradient of CD11c^+^ MOIC density in three-month-old mice (nasal: 1003 ± 369 cells/mm^2^, vs. central: 946 ± 574 cells/mm^2^, vs. temporal: 731 ± 353 cells/mm^2^, *p* = 0.044). Cx3cr1-deficient mice had two-fold fewer MHC-II^+^ MOICs, suggesting a role for Cx3cr1 receptor signaling in meibomian gland surveillance. CD11c^+^ MOIC density was lower in BAK-exposed eyes compared to saline-treated controls, suggesting a change in homeostasis. This study provides novel insight into resident ICs located at MGOs, and their contribution to MG homeostasis.

## 1. Introduction

Meibomian glands (MGs) are modified sebaceous glands that are located within the tarsal plate of the eyelids [1,2]. These holocrine glands secrete meibum, an oil-rich substance, onto the ocular surface, contributing to the outer lipid layer of the tear film [3,4]. Meibomian gland orifices (MGOs) are located at the interface between the external environment of the eyelid and the inner luminal duct that expels meibum onto the ocular surface [4,5,6]. Heterogeneity of MG function exists depending on anatomical location, with glands in the nasal eyelid margin reported to be more active than temporally located glands in humans [7]. In addition, it has been proposed that gland activity is asynchronous, with not all glands actively secreting meibum at the same time [8]. 

Meibomian gland dysfunction (MGD) is characterised by gland atrophy, ductal hyperkeratinisation, obstruction, and inflammation, which perturb the production and secretion of meibum [4]. Investigations into human tissue specimens of the meibomian gland found greater CD45^+^ leukocyte infiltration into the acini when meibum expression grade was more severe [9]. In vivo confocal microscopy (IVCM) of purported immune cells (ICs) in the palpebral conjunctiva and openings of acinar ducts in patients with MGD had previously been shown to have a positive correlation with worse symptom scores and shorter tear evaporation times [10]. These studies indicate that compromise to the secretory function of the meibomian glands affects the integrity of the tear lipid layer, which can lead to the evaporative form of dry eye disease [11]. The causes of dry eye disease (DED) are multifactorial, and include ageing, metabolic disturbances, autoimmune disease, contact lens wear, and use of certain systemic and topical medications [4]. In addition to ocular surface symptoms of irritation and discomfort, DED induces a range of clinical signs that include tear hyperosmolarity and ocular surface inflammation [12]. Studies investigating MGD have primarily focused on acinar units that are situated deeper within the gland. However, less is known about the IC characteristics around the MGOs that associate more closely with the ocular surface and tear film perturbations found in MGD.

DED often has an inflammatory connection, and thus changes might be expected to affect resident ICs in ocular surface structures. The palpebral conjunctiva hosts a diverse range of resident ICs within conjunctiva-associated lymphoid tissue that is distributed more densely in the tarsal and orbital regions with varying distribution in each geographical region of the eyelid [13]. Individuals with MGD have four-fold greater density of inflammatory ICs in the palpebral conjunctival epithelium compared to those without the disease [10]. Specific antigen-presenting ICs, known as dendritic cells (DCs), enable communication between the innate and adaptive immune systems during inflammation [14]. DC maturation and communication with T cells during inflammatory states in the conjunctiva are enabled by the chemokine receptor Cx3cr1 on the cell surface of DCs, whose cognate protein is Cx3cl1, also known as fractalkine [15]. Conjunctival expression of Cx3cl1 is known to be increased by the use of topical glaucoma medications preserved with benzalkonium chloride (BAK) [15]. BAK is a common preservative used in topical medications, and is known to be cytotoxic to corneal epithelial cells [16]. Cx3cr1-Cx3cl1 signalling is also critical for the migration of MHC class II^+^ (MHC-II^+^) ICs to the mouse corneal epithelium [17]. CD11c and MHC-II molecules are also on the cell surface of DCs and macrophages, with MHC-II expression important for antigen presentation to T cells [18]. CD11c^+^ DCs with extended processes have recently been found to be closely related to goblet cells in the conjunctiva, and to also upregulate MHC-II expression in conditions of increased microbial load [19]. This indicates the role of DCs in surveying the conjunctival epithelium and the importance of resident ICs in maintaining ocular surface homeostasis. Whether the characteristics of these ICs are altered in inflammatory states is yet to be investigated. 

Current understanding of the immunological profile of MGs mostly stems from animal studies of MGD, with a focus on the pathophysiology of the acinar units [5,20]. In aged mice, acinar atrophy is associated with an increase in CD45^+^ bone marrow-derived ICs [21] and F4/80^+^ macrophages [22,23,24]. The accumulation of neutrophil extracellular traps within the MG lumens of mice with allergic eye disease, and in conjunctival biopsies from patients with trichiasis, provides evidence of a role for inflammatory cells in MG obstruction [25]. However, little is known about the resident IC populations that are associated with MGOs along the eyelid margin in healthy young mice and whether they are affected by age. 

Given the paucity of information about IC populations associated with MGOs, and a potential relationship with MGD and DED, the present study sought to investigate the distribution, density, and phenotype of IC populations residing around healthy MGOs, which are termed meibomian gland orifice-associated immune cells (MOICs), in three-month-old and seven-month-old mice. Specifically, CD11c^+^ DCs, Cx3cr1^+^ ICs, and MHC-II^+^ ICs were the primary focus of this investigation. As the chemokine receptor Cx3cr1 has been shown to regulate the presence of MHC-II^+^ ICs in the corneal epithelium [17], the effects of the Cx3cr1 receptor on the number and distribution of MHC-II^+^ MOICs in the normal mouse eyelid were also investigated. The influence of topical BAK exposure on the presence and morphology of CD11c^+^ MOICs was also investigated.

## 2. Results

### 2.1. CD11c^+^ MOICs

#### 2.1.1. CD11c^+^ MOICs Surround MGOs along the Eyelid Margin

**Figure 1:**
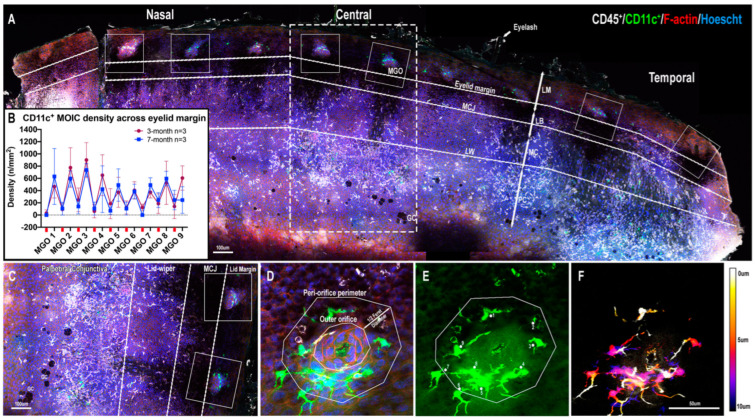
Low-power view of eyelid flatmount showing imaging approach. (**A**) Two adjacent MGOs in each eyelid region (nasal, central, and temporal) were imaged using confocal microscopy. (**B**) Graphical depiction of CD11c^+^ MOIC density across a typical eyelid margin including MGO and inter-MGO spaces (red dashes on X-axis). (**C**) An enlarged image of the central eyelid (dotted box in A). (**D**) MOICs surrounding an MGO highlighted by F-actin (red). Peri-orifice area is calculated by adding half the Feret diameter of the outer orifice. (**E**) CD11c^+^ MOICs within the peri-orifice area were manually counted. (**F**) Depth-colour projection of CD11c^+^ MOICs around an MGO of (**E**) where cooler colours represent MOICs located deeper in the duct and warmer colours indicate cells closer to the epithelial surface. Dendrites can extend towards the epithelium and deeper to 10 μm. The same scale bar in (**F**) applies to (**D**,**E**). Lid margin, LM, lid margin border, LB, marginal conjunctiva, MC, palpebral conjunctiva, PC, mucocutaneous junction, MCJ, lid-wiper area, LW, goblet cells, GC, meibomian gland orifice, MGO.

Eyelid margin flatmounts revealed clusters of CD11c^+^ MOICs in both sexes and both age groups in each region (Figure 1A). The density of CD11c^+^ MOICs was greater around MGOs compared to the inter-glandular spaces between the MGOs (Figure 1B). This can be clearly seen in a magnified image of two central MGOs (Figure 1C). Occasionally, when two adjacent orifices were compared within a region there was a large variability in CD11c^+^ MOIC density, whereby one MGO would have significantly fewer CD11c^+^ MOICs than its immediate neighbour (Figure S1A–C). To visualise the vertical distribution of MOICs from the eyelid surface towards the ducts, colour-coded depth projections were generated, with MOICs appearing to be evenly distributed throughout the ducts (Figure 1D–F). 

#### 2.1.2. Decreasing Distribution Gradient of CD11c^+^ MOIC Density across the Eyelid Margin in Three-Month-Old Mice

**Figure 2:**
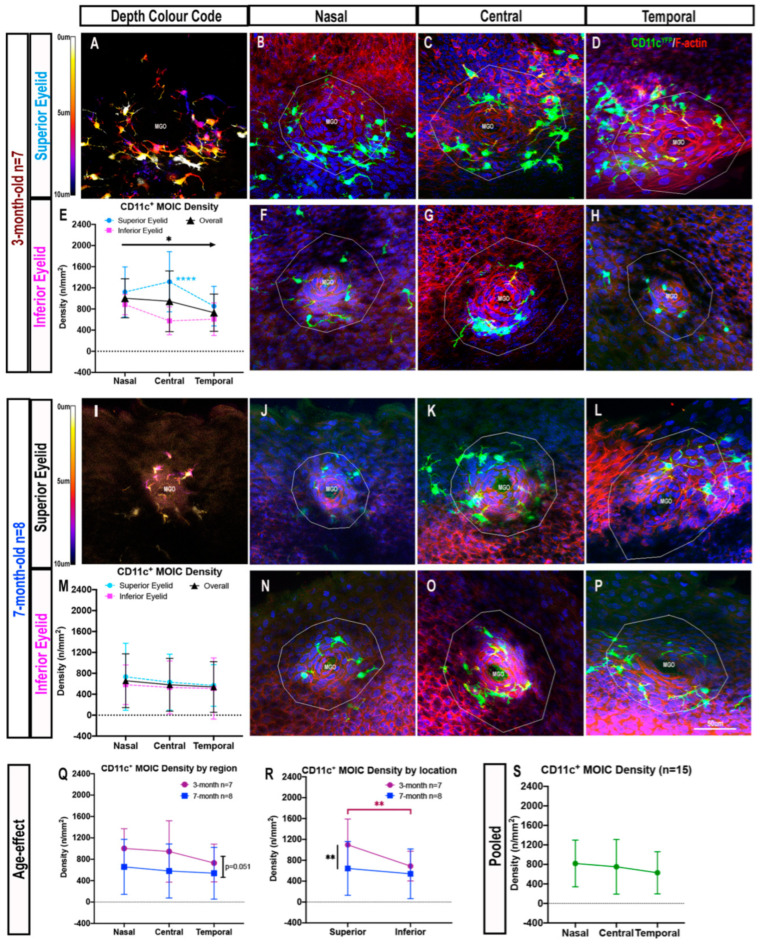
CD11c^+^ MOICs in each eyelid region in three-month-old (**A**–**H**) and seven-month-old (**I**–**P**) mice. Depth colour code representation of CD11c^+^ MOICs surrounding an MGO in three-month-old (**A**) and seven-month-old (**I**) mice. CD11c^+^ MOICs surrounding MGO in the superior and inferior eyelids, three-month-old: (**B**,**F**) nasal, (**C**,**G**) central, (**D**,**H**) temporal; seven-month-old: (**J**,**N**) nasal, (**K**,**O**) central, (**L**,**P**) temporal. Graphs representing CD11c^+^ MOIC density across the superior and inferior lids in three-month-old (**E**) and seven-month-old (**M**) mice. (**R**) Graph depicting the effect of age on CD11c^+^ MOIC distribution by eyelid region (**Q**) and location (i.e., superior and inferior). (**S**) Overall CD11c^+^ MOIC distribution after pooling data from both age groups. All data are presented as mean ± SD, * *p* ≤ 0.05; ** *p* ≤ 0.01; **** *p* ≤ 0.0001. The same scale bar in (**P**) applies to all confocal images.

In three-month-old mice, CD11c^+^ MOICs were abundant in the superior (Figure 2A–D) and inferior eyelid margins (Figure 2F–H). When considering the effect of eccentricity differences by pooling superior and inferior eyelid data, there was a decreasing density gradient of CD11c^+^ MOICs from nasal (1003±369 cells/mm^2^) to central (946±574 cells/mm^2^) and temporal MGOs (731 ± 353 cells/mm^2^, *p* = 0.044) (Figure 2E). When comparing CD11c^+^ MOIC density in the six distinct regions across the eyelid margin, there was only a significant difference in the central location between the superior and inferior eyelids (superior nasal: 1122 ± 474 cells/mm^2^ vs. inferior nasal: 884 ± 193 cells/mm^2^; *p* > 0.05, superior central: 1316 ± 569 cells/mm^2^ vs. inferior central: 576 ± 265 cells/mm^2^, *p* < 0.0001; superior temporal: 854 ± 374 cells/mm^2^ vs. inferior temporal: 608 ± 308 cells/mm^2^, *p* > 0.05) (Figure 2E). 

In seven-month-old mice, CD11c^+^ MOIC cells were visible in the superior eyelid (Figure 2I–L) and inferior eyelid (Figure 2N–P). With respect to eyelid region, pooled data from both superior and inferior eyelids revealed similar MOIC densities across the eyelid (nasal: 658±514 cells/mm^2^ vs. central: 581 ± 506 cells/mm^2^ vs. temporal: 539 ± 484 cells/mm^2^, *p* > 0.05; Figure 2M). When comparing age groups by eyelid region, there were no inter-group age differences in mean CD11c^+^ MOIC density in any region (*p* = 0.051) (Figure 2Q). When considering superior and inferior eyelid location, three-month-old mice had a greater density of CD11c^+^ MOICs than seven-month-old mice (893 ± 449 cells/mm^2^ vs. 593 ± 493 cells/mm^2^, respectively, *p* = 0.004) (Figure 2R). Multiple comparisons revealed this difference to be mostly attributable to IC densities in the superior eyelid (three-month-old: 1097 ± 494 cells/mm^2^ vs. seven-month-old: 644 ± 515 cells/mm^2^, *p* = 0.003), but not in the inferior eyelid (three-month-old: 689 ± 284 cells/mm^2^ vs. seven-month-old: 541 ± 476 cells/mm^2^, *p* > 0.05) (Figure 2R). Intra-group comparisons also revealed that three-month-old mice had a higher density of CD11c^+^ MOICs in the superior eyelid (1097 ± 494 cells/mm^2^) compared to the inferior eyelid (689 ± 284 cells/mm^2^, *p* = 0.009) (Figure 2R). This difference was not observed in seven-month-old mice (superior: 644 ± 515 cells/mm^2^ vs. inferior: 541 ± 476 cells/mm^2^, *p* > 0.05) (Figure 2R). 

In terms of regional differences across the eyelid margin (nasal, central, and temporal), irrespective of age or eyelid location (superior vs. inferior), the CD11c^+^ MOIC density from nasal to temporal regions was similar (nasal: 819 ± 478 cells/mm^2^ vs. central: 751 ± 560 cells/mm^2^ vs. temporal: 628 ± 432 cells/mm^2^, *p* > 0.05, Figure 2S).

There were no sex differences when considering CD11c^+^ MOICs in each anatomical location (*p* > 0.05, data not shown).

### 2.2. Cx3cr1^+^ MOIC Density Is Lower Than CD11c^+^ MOIC Density

**Figure 3:**
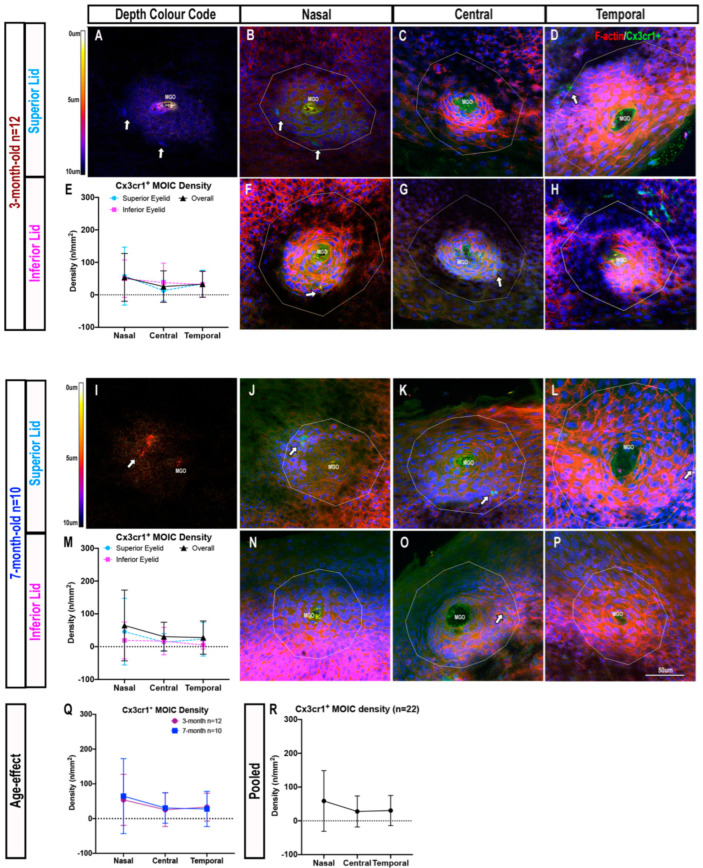
Cx3cr1^+^ MOICs surrounding MGOs in each eyelid region in three-month-old (**A**–**H**) and seven-month-old (**I**–**P**) mice. Depth colour code of Cx3cr1^+^ ICs surrounding an MGO in three-month-old (**A**) and seven-month-old (**I**) mice. Cx3cr1^+^ MOICs tend to be located deeper in the MG, and have shorter and fewer dendrites. Cx3cr1^+^ MOICs in the superior and inferior eyelids, three-month-old: (**B**,**F**) nasal, (**C**,**G**) central, (**D**,**H**) temporal; seven-month-old: (**J**,**N**) nasal, (**K**,**O**) central, (**L**,**P**) temporal. White arrows are Cx3cr1^+^ MOICs. Cx3cr1^+^ MOIC density across the superior and inferior lids in three-month-old (**E**) and seven-month-old (**M**) mice, and no effect of age on Cx3cr1^+^ MOIC distribution by eyelid region (**Q**). Overall Cx3cr1^+^ MOIC density distribution by region (**R**), pooling data from both age groups. All data are presented as mean ± SD. The same scale bar in (**P**) applies to all confocal images.

Immunofluorescence imaging revealed the overall density of Cx3cr1^+^ MOICs in both age groups to be lower than CD11c^+^ MOICs, with cells tending to be situated deeper in the MGOs (Figure 3A,I). Cx3cr1^+^ MOICs appeared less dendriform and the fluorescent signal was less prominent than CD11c eYFP^+^ cells (Figure 3B–D, F–H, J–L, N–P and Figure S2). Figure S2 contains single channel images of Cx3cr1^+^ cells, to demonstrate their faint, but positive, signal in the absence of the F-actin and Hoechst channels.

In three-month-old mice, Cx3cr1^+^ MOICs were evenly distributed across the eyelid (nasal: 54 ± 74 cells/mm^2^ vs. central: 25 ± 48 cells/mm^2^ vs. temporal: 33 ± 40 cells/mm^2^, *p* > 0.05). Cell densities in the superior and inferior eyelids were also similar (superior: 35 ± 61 cells/mm^2^ vs. inferior: 40 ± 52 cells/mm^2^, *p* > 0.05, Figure 3A–H). Similarly, in seven-month-old Cx3cr1^gfp/gfp^ mice, there were no significant differences in Cx3cr1^+^ MOIC density in relation to eyelid region (nasal: 65 ± 108 cells/mm^2^ vs. central: 31 ± 44 cells/mm^2^ vs. temporal: 28 ± 51 cells/mm^2^, *p* > 0.05) or eyelid location (superior: 55 ± 33 cells/mm^2^ vs. inferior: 27 ± 15 cells/mm^2^, *p* > 0.05, Figure 3I–P). When considering mean MOIC density for eyelid regions, irrespective of superior or inferior locations, there were no intergroup age differences in Cx3cr1^+^ MOIC density (*p* > 0.05, Figure 3Q). Overall, the density of Cx3cr1^+^ MOICs in three-month-old mice (37 ± 15 cells/mm^2^) was similar to seven-month-old mice (41 ± 21 cells/mm^2^, *p* > 0.05, Figure 3Q). When only region was considered, by combining data across ages, there was no difference in Cx3cr1^+^ MOIC density from the nasal to temporal eyelid region (nasal: 59 ± 90 cells/mm^2^ vs. central: 28 ± 46 cells/mm^2^ vs. temporal: 30 ± 45 cells/mm^2^, *p* > 0.05, Figure 3R).

### 2.3. The Presence of MHC-II^+^ MOICs Is Partially Dependent on the Cx3cr1 Receptor

**Figure 4:**
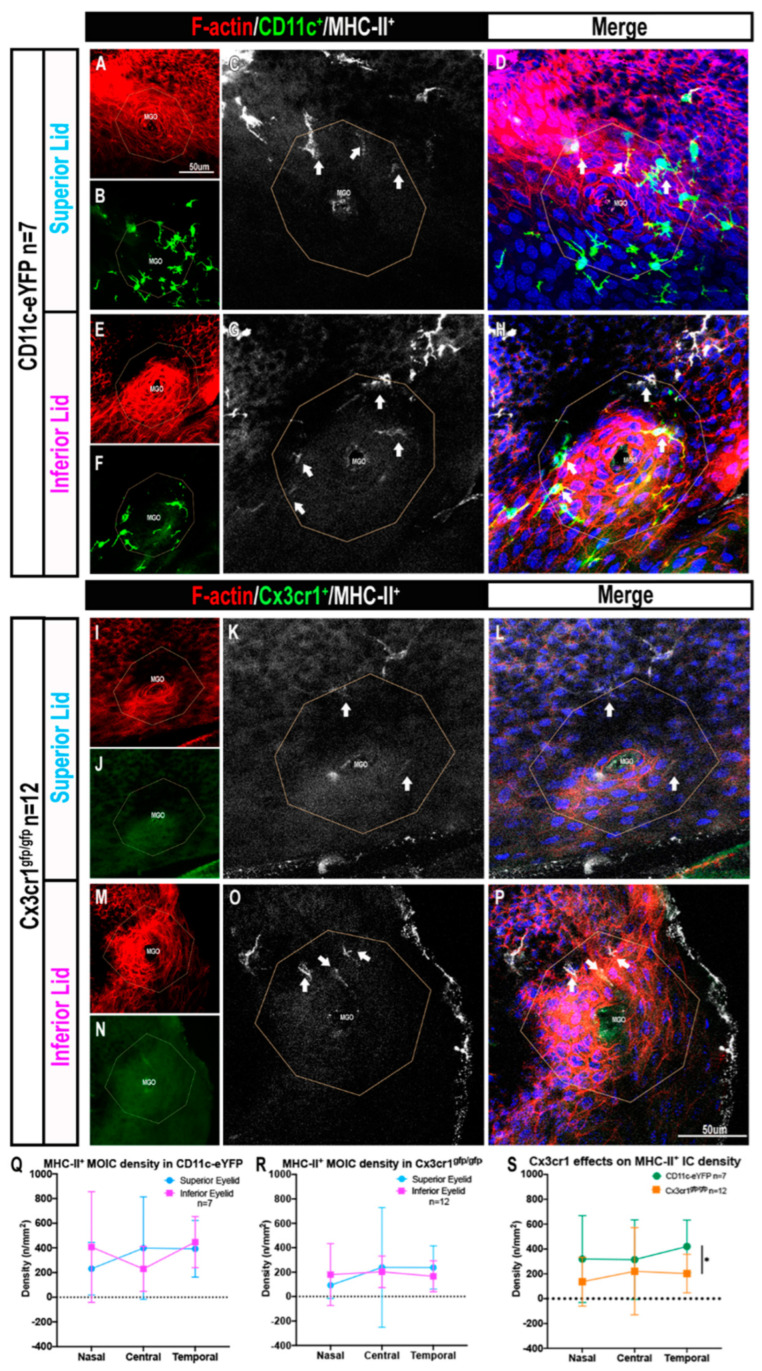
Confocal microscopy images of MHC-II^+^ MOICs. F-actin (red) highlighting the MGO in CD11c-eYFP mice; superior lid (**A**–**D**) and inferior lid (**E**–**H**), and Cx3cr1^gfp/gfp^ mice; superior lid (**I**–**L**) and inferior lid (**M**–**P**). White arrows are MOICs that are MHC-II^+^. Graph represents MHC-II^+^ MOIC distribution in CD11c-eYFP (**Q**) and Cx3cr1^gfp/gfp^ (**R**) mice and the effects of Cx3cr1 deficiency on MHC-II^+^ MOIC density along the eyelid margin (**S**). All data are presented as mean ± SD, (* *p* ≤  0.05). Scale bar in (**A**) applies to panels (**B**,**E**,**F**,**I**,**J**,**M**,**N**), and in (**P**) applies to panels (**C**,**D**,**G**,**H**,**K**,**L**,**O**,**P**).

In CD11c^eYFP^ mice, the density of MHC-II^+^ MOICs was similar in the superior (Figure 4A–D) and inferior (Figure 4E–H) eyelids (superior: 341 ± 296 cells/mm^2^ vs. inferior: 361 ± 304 cells/mm^2^, *p* > 0.05, Figure 4Q). MHC-II^+^ MOICs were evenly distributed in all eyelid regions (nasal: 319 ± 349 cells/mm^2^ vs. central: 314 ± 320 cells/mm^2^ vs. temporal: 420 ± 212 cells/mm^2^, *p* > 0.05; Figure 4Q). 

In Cx3cr1^gfp/gfp^ (deficient) mice, MHC-II^+^ MOICs were observed in the superior (Figure 4I–L) and inferior eyelids (Figure 4M–P). Quantitative analysis revealed no differences in MHC-II^+^ MOIC density across the eyelid regions (nasal: 137 ± 196 cells/mm^2^ vs. central: 221 ± 351 cells/mm^2^ vs. temporal: 202 ± 155 cells/mm^2^, *p* > 0.05) and eyelid location (superior: 190 ± 307 cells/mm^2^ and inferior 183 ± 175 cells/mm^2^, *p* > 0.05, Figure 4R).

Overall, when comparing the density of MHC II^+^ ICs in the two mouse strains, there were approximately two-fold fewer MHC-II^+^ MOICs in Cx3cr1^gfp/gfp^ mice (187 ± 248 cells/mm^2^) than in CD11c^eYFP^ mice (351 ± 297 cells/mm^2^, *p* = 0.010, Figure 4S).

### 2.4. MOICs Express CD11c and Cx3cr1 in CD11c-eYFP Mice

Multicolour flow cytometry analysis of the mouse eyelid margin demonstrated a greater mean percentage of CD45^+^Cx3cr1^+^ MOICs in CD11c^eYFP^ mice compared with Cx3cr1^gfp/gfp^ mice (CD11c^eYFP^: 38.2 ± 7.0% vs. Cx3cr1^gfp/gfp^: 3.1 ± 0.5%) (Figure S5(Bi)). The percentage of double positive CD45^+^CD11c^+^ MOICs in both strains of mice was similar (CD11c^eYFP^: 5.2 ± 3.5% vs. Cx3cr1^gfp/gfp^: 3.3 ± 1.2%, mean ± SD) (Figure S5(Bii)). In CD11c^eYFP^ mice, the percentage of CD45^+^CD11c^+^ (5.2 ± 3.5%) and CD45^+^CD11c^+^Cx3cr1^+^ MOICs (4.8 ± 5.5%) was similar (Figure S5(Bii) vs. (iii)), suggesting that most CD11c^+^ cells likely also expressed Cx3cr1 in mice with functional Cx3cr1 receptor expression. The percentage of triple positive CD45^+^Cx3cr1^+^MHC-II^+^ MOICs were two-fold greater in CD11c^eYFP^ mice compared to Cx3cr1^gfp/gfp^ mice (CD11c^eYFP^: 2.6 ± 1.4% vs. Cx3cr1^gfp/gfp^: 1.2 ± 0.5), however, statistical analysis was not possible due to the need to pool animals. The percentage of CD45^+^CD11c^+^MHC-II^+^ MOICs (CD11c^eYFP^: 0.8 ± 0.6% vs. Cx3cr1^gfp/gfp^: 0.7 ± 0.3%) was similar in both strains of mice (Figure S5(Biv) and (v), respectively). 

### 2.5. Topical Benzalkonium Chloride (BAK) Reduces CD11c^+^ MOIC Density

**Figure 5:**
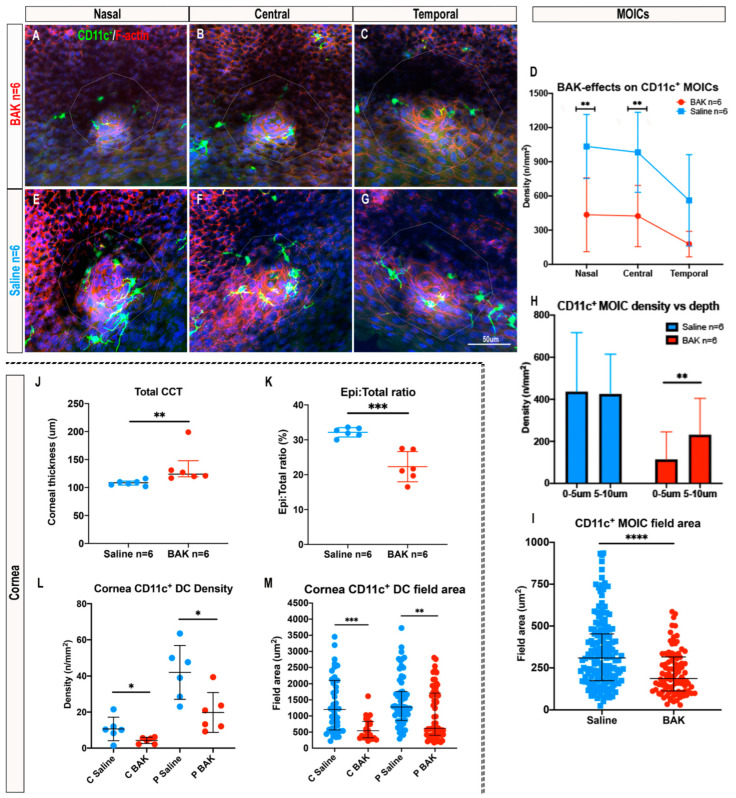
BAK- vs. saline-treated eyes. Confocal microscopy images of CD11c^+^ MOICs around MGOs in BAK-treated CD11c-eYFP mouse eyes (**A**–**C**) and saline-treated controls (**E**–**G**). (**D**) Graph represents the effect of BAK on CD11c^+^ MOICs in each region, and the distribution of CD11c^+^ MOICs from 0–5 µm and 5–10 µm depths in saline controls vs. BAK-treated eyes (**H**), mean ± SD. (**I**) CD11c^+^ MOIC field area in saline controls vs. BAK-treated eyes, median (inter-quartile-range). (**J**) Total central corneal thickness (CCT) between saline controls and BAK-treated eyes, median (inter-quartile-range). (**K**) Corneal epithelial thickness: total central corneal thickness ratio (%) between saline controls and BAK-treated eyes. (**L**) Corneal CD11c^+^ DC density in the central and peripheral corneal regions in saline controls vs. BAK-treated eyes. (**M**) Corneal CD11c^+^ DC morphology, measured by field area, in the central and peripheral corneal regions comparing saline controls vs. BAK-treated eyes, median [inter-quartile-range]. For (**L**) and (**M**): C, central; P, peripheral. For statistical analyses: * *p* ≤  0.05; ** *p* ≤  0.01; *** *p* ≤  0.001; **** *p* ≤  0.0001. Scale bar in (**G**) applies to all confocal images.

Overall, BAK-exposed eyes (Figure 5A–C) had a 2.5-fold lower CD11c^+^ MOIC density than saline-treated controls (Figure 5G) (saline: 859 ± 393 cells/mm^2^ vs. BAK: 346 ± 266 cells/mm^2^, *p* = 0.005, Figure 5D). Multiple comparisons revealed these differences were in the nasal (saline: 1034 ± 281 cells/mm^2^ vs. BAK: 435 ± 324 cells/mm^2^, *p* = 0.006) and central (saline: 983 ± 351 cells/mm^2^ vs. BAK: 424 ± 268 cells/mm^2^, *p* = 0.010) eyelid regions, but were not evident temporally (saline: 561 ± 400cells/mm^2^ vs. BAK: 178 ± 112 cells/mm^2^, *p* > 0.05) (Figure 5D). Eyes treated with BAK did not have regional differences in CD11c^+^ MOIC density across the eyelid margin (*p* = 0.056), whereas saline-treated controls maintained the nasal–temporal decreasing gradient of MOICs (nasal vs. temporal, *p* = 0.015, central vs. temporal, *p* = 0.028, Figure 5D), similar to that observed in three-month-old naïve mice (see Section 2.1.2).

When considering the location of MOICs with respect to distance from the surface epithelium, in eyes treated with BAK, denser CD11c^+^ MOICs were located deeper in the MGO (0–5 µm: 114 ± 132 cells/mm^2^ vs. 5–10 µm: 232 ± 173 cells/mm^2^, *p* = 0.006) (Figure 5A–C, orange columns in Figure 5H and Figure S3A). This difference was not evident in saline controls where CD11c^+^ MOICs were evenly distributed throughout the 10 µm depth of the MGO (0–5 µm: 437 ± 280 cells/mm^2^ vs. 5–10 µm: 425 ± 189 cells/mm^2^, *p* > 0.05) (Figure 5E–G, blue columns Figure 5H and Figure S3B). Changes to IC morphology, which are an indicator of cell activation in ocular surface DCs [26,27], were quantified to further evaluate the inflammatory effects of topically applied BAK. On average, MOICs in BAK-treated eyelids had smaller field areas than saline controls, (median (inter-quartile range: BAK: 187 (586–113) µm^2^ vs. saline: 309 (453–175) µm^2^, *p* < 0.0001) (Figure 5I)).

The inflammatory effects of BAK were also confirmed by assessing central corneal thickness (CCT) via optical coherence tomography (OCT). CCT in BAK-treated eyes (median (inter-quartile range): 124 (148–119) mm) was thicker than in saline controls (109 (112–104) mm), *p* = 0.002 (Figure 5J). In the BAK-exposed eyes, the ratio (%) of the epithelial thickness to total CCT was lower, consistent with the presence of corneal edema due to an increase in stromal thickness (BAK: 22 ± 4% vs. saline: 32 ± 1%, *p* = 0.0003, Figure 5K). 

Similar characteristics in the MGs were evident in the cornea, with corneal CD11c^+^ DC density lower in BAK-treated eyes compared with saline controls in the central (BAK: 4 ± 2 cells/mm^2^ vs. saline: 11 ± 7 cells/mm^2^, *p* = 0.044) and peripheral (BAK: 20 ± 11 cells/mm^2^ vs. saline: 42 ± 15 cells/mm^2^, *p* = 0.015) corneal regions (Figure 5L). Corneal CD11c^+^ DCs also had smaller field area in BAK-exposed eyes in both the central (BAK: 543 (830–323) mm^2^ vs. saline: 1201 (2102–568) mm^2^, *p* = 0.0002) and peripheral cornea (BAK: 616 (1711–403) mm^2^ vs. saline: 1278 (1759–860) mm^2^, *p* = 0.0011) (Figure 5M and Figure S3C,D).

## 3. Discussion

MGOs enable the secretion of meibum onto the eyelid margin, where it integrates with multiple constituents to form a component of the tear film. MGOs are constantly exposed to external factors, including potential pathogens, at the ocular surface [4,5,11]. Given the inflammatory nature of ocular surface diseases such as DED, it is well understood that the corneal and conjunctival epithelium have a population of resident ICs that survey these tissues to maintain homeostasis and that these cells are disrupted in dry eye conditions. These include DCs and MHC-II^+^ antigen-presenting cells in both tissues [1,17], as well as CD8^+^, CD4^+^, and memory T cells in the conjunctiva [13,28,29]. MGD is also inflammatory in nature and one of the main contributors to evaporative DED [5]. However, less is understood regarding resident ICs and their role in maintaining homeostasis of the meibomian glands. To date, the presence of ICs around MGOs has not been described. The current study is the first, to our knowledge, to quantify and phenotypically characterise IC populations that reside around MGOs and to consider the effect of inflammation on the distribution and morphology of MOICs. 

Of the IC phenotypes examined using immunofluorescence staining, the predominant cell types around the MGOs were identified as CD11c^+^ (presumed DCs), followed by MHC-II^+^ ICs. To consider the repertoire of cell surface molecules expressed by these cells, flow cytometry was performed using carefully dissected eyelid margins from both strains of mice. Flow cytometry results confirm that CD11c^+^ cells extracted from the eyelid co-expressed Cx3cr1, indicating that the most likely phenotype of MOICs is CD11c^+^Cx3cr1^+^ cells in normal mouse eyelids. The number of Cx3cr1^gfp/gfp^ cells was lower in mice lacking Cx3cr1 compared to the number of Cx3cr1^+^ cells identified in the CD11c^eYFP^ mice, suggesting that Cx3cr1 deficiency influences the density of MOICs. A caveat to these experiments is that it was not possible to isolate pure populations of MOICs from the surface of the eyelid margin, and thus it is likely that immune cells located deep to the marginal eyelid epithelium were included. Another limitation was that the pooling of samples (due to small tissue volumes and low cell numbers) prevented statistical comparisons from being performed. 

Typically, CD11c^+^ MOICs and MHC-II^+^ MOICs were observed to cluster around MGOs and were less frequent in the inter-glandular spaces; this may indicate that they function to support the epithelial ducts, and/or that ductal epithelial cells express chemokines that attract these ICs to the orifices. Furthermore, CD11c^+^ MOICs were distributed consistently throughout the 10 µm depth of the MGO surface. The presence of these orifice-associated ICs aligns with reports in other murine exocrine glands that harbour ductal macrophages and DCs [30] which have been proposed to function in immune surveillance and antigen presentation, as well as phagocytosis of debris in epithelial ducts [30]. In the mammary glands of mice, CD11c^+^ DCs and MHC-II^+^ ICs actively monitor and regulate morphogenesis of the mammary epithelium and ducts, particularly during alveoli involution post-lactation [31,32]. In murine salivary gland ducts, resident ductal-associated ICs assist memory T cells in maintaining tissue homeostasis, and cellular recruitment into the inflamed gland [33]. While the present study reports on the presence and distribution of MOICs in the eyelids of three-month-old and seven-month-old mice, the precise function of these cells is unknown, and requires further investigation. 

In the corneal epithelium, resident CD11c^+^ DCs contribute to corneal nerve maintenance and re-innervation after injury [34,35,36]. The distribution of epithelial CD11c^+^ DCs in the cornea is well described, with a greater density of cells in the peripheral cornea (in closer proximity to the vascular limbus), compared to the central cornea [37,38]. Given the known gradients of IC density in the corneal epithelium, we investigated whether the density of MOICs was influenced by eyelid location (superior vs. inferior) or region (nasal vs. central vs. temporal). Interestingly, there was a greater density of CD11c^+^ MOICs in the nasal eyelid region compared to the temporal region in three-month-old mice. We speculate this may relate to the natural action of blinking, whereby the motion of both eyelids pushes tear film debris and contents towards the nasal punctum [39]. This region, therefore, may require greater immune surveillance of the orifices as it is exposed to more pathogens. We also observed substantial variability in the density of MOICs in immediately adjacent MGOs. While the reason(s) for this variation is unclear, a prior clinical study reported that MG activity and meibum secretion were not synchronous across all glands, and that nasally located MGs were more active than those in the central or temporal eyelid regions [8]. Whether disparities in the density and distribution of MOICs across the eyelid relate to the activity of individual MGs is yet to be determined.

Differential distributions and densities of conjunctival ICs have been reported previously. Knop and Knop (2002) reported a greater density of conjunctival-associated lymphoid tissue in the tarsal and orbital regions of the conjunctiva [40], with larger areas of conjunctival crypts in the tarsal region [41]. A study in healthy human eyes investigated the topographical distribution of intraepithelial lymphocytes, demonstrating a decreasing density gradient of CD45^+^ ICs from the superior tarsal conjunctiva to the inferior tarsal–bulbar–fornix, and upper bulbar conjunctiva [42]. The same study also reported a greater proportion of CD3^+^ and CD8^+^ T cells in the superior tarsal and bulbar conjunctiva, whereas the inferior tarsal–bulbar–fornix had a higher number of CD19^+^ B cells based on flow cytometry analysis. 

With respect to regional differences in other cellular features on the ocular surface, a recent study reported a decreasing nasal–temporal gradient in goblet cell density in the lower eyelid of mice; the opposite effect was observed in the superior eyelid [43]. In the present study, when considering the distribution of MOICs along the eyelid margin, there was a decreasing nasal–temporal density gradient of CD11c^+^ MOICs in 3-month-old mice. This pattern was not evident in seven-month-old mice, who had fewer CD11c^+^ MOICs across all eyelid regions. These findings suggest subtle age-related changes that may alter the density of MOICs in each region, however, the mechanism(s) underlying these changes require further investigation. In other areas of the conjunctiva, CD11c^+^ DCs and MHC-II^+^ ICs are found in higher numbers in aged mice (i.e., 24 months old), particularly in the basal layer of the palpebral conjunctiva [44]. However, an earlier study on healthy human conjunctival tissue specimens found the density of Langerhans cells (a subset of DCs) decreased with age from 4.4 cells/mm^2^ (≤20 years) to 1.2 cells/mm^2^ (>60 years), in the palpebral (central), bulbar (superior lateral), and forniceal (central) regions [45]. In the present study, we found an age-related plateauing of CD11c^+^ MOICs density gradient, although relatively young mice were used in comparison to the previous studies. Future studies should compare the density of MOICs in older mice (i.e., 12 to 24 months of age). Furthermore, although the sample size of each sex was low, statistical analysis revealed no differences in MOIC density between sexes in both age groups. In humans, the prevalence of meibomian gland dysfunction is greater in women over the age of 40 years [46,47]. Knowledge of the presence and distribution of MOICs will inform future studies, which may examine the influence of age and sex on these cells around the MGOs.

Fewer Cx3cr1^+^ MOICs were found in Cx3cr1-deficient mice compared with CD11c^eYFP^ mice that have normally functioning Cx3cr1 receptors. Furthermore, our immunofluorescence and flow cytometry data indicate a partial contribution of Cx3cr1 in the presence of MHC-II^+^ MOICs, as evidenced by fewer MHC-II^+^ MOICs in Cx3cr1-deficient mice. These findings are similar to the reported dependence of Cx3cr1 on the homing of MHC-II^+^ ICs to the corneal epithelium of healthy mice [17]. In the gut, Cx3cr1 signaling is required for the formation of transepithelial dendrites in DCs to sample intestinal luminal antigens [48,49]. In the steady-state mouse conjunctival epithelium, Cx3cr1 deficiency does not influence the presence of resident IC populations (i.e., Cx3cr1^+^, CD11b^+^, F4/80^+^, CD3^+^, and NKG2D^+^) but is associated with lower inflammatory cell infiltration following acute topical exposure to BAK (0.05%, 15 drops in one hour) [15]. Whether Cx3cr1 affects the homeostatic recruitment of other CD45^+^ MOICs, rather than only the Cx3cr1^+^ and MHC-II^+^ population, is yet to be determined and will be the focus of a subsequent investigation. 

Animal and tissue culture studies highlight that eye drops with preservatives, such as BAK-preserved glaucoma medication, have cytotoxic side effects on the cornea and conjunctiva [16]. The breakdown of the corneal epithelial lipidome [50], reduced cell viability [51,52], and increased conjunctival subepithelial inflammation [53] are known effects of BAK cytotoxicity. Clinical indicators include ocular discomfort and increased corneo-conjunctival staining [54]. Ocular surface exposure to BAK increases the risk of diseases including DED and MGD in humans [55], where both conditions contribute to a pro-inflammatory environment [11]. In vivo confocal microscopy imaging of the conjunctiva in humans demonstrates that patients with DED have a greater density of ICs in the palpebral conjunctival stroma and epithelium [10]. Bright hyperreflective cells surrounding, and within, the MG ducts have also been described, however, the phenotype of these cells is not known [10]. In a clinical study, Zhivov et al. (2010) exposed healthy human eyes to 0.01% BAK three times daily for 12 weeks and reported a significant increase in DCs in the central corneal epithelium [56]. 

To test whether daily topical application of 0.1% BAK would affect MOIC density, corneal and eyelid flatmounts of CD11c^eYFP^ reporter mice were examined after exposure and compared to saline-treated controls. In contrast to the aforementioned corneal and conjunctival findings, BAK-treated eyes showed an overall lower density of CD11c^+^ MOIC and corneal CD11c^+^ DCs. These CD11c^+^ MOICs were smaller (based on field area analysis) and appeared to have a greater density deeper along the MG duct compared to superficially. Corneal epithelial CD11c^+^ DCs followed a similar pattern with smaller field area and lower density in both central and peripheral regions in BAK-treated eyes. The inflammatory effects of BAK were confirmed by a thicker CCT and lower corneal epithelial:total thickness ratio, consistent with the presence of corneal oedema. Whether the CD11c^+^ MOICs retract their dendrites and migrate into the ductal regions of the gland, or if the cells proximal to the MGO are undergoing apoptosis in response to BAK exposure, remains to be investigated. 

The current study compared three-month-old with seven-month-old mice. The age of these mice equates to approximately five, and 20 to 35, human years, respectively (calculated from [57,58]). Whilst the age cohorts in this study might be considered relatively young in human years, there were more CD11c^+^ MOICs in the three-month-old compared to seven-month-old mice, with only subtle changes in Cx3cr1^+^ MOICs. Future studies comparing MOICs in mice aged 12 to 24 months, which are more representative of the population in humans who suffer from meibomian gland dysfunction (>40 years [47]), would be beneficial to extend these findings. Additionally, the BAK model used in the current study involved a concentration of 0.1%, administered topically once daily via eye drops for seven days, which is a higher dose than in most topical medications. Treatment for an extended period with a lower dosage may more closely reflect chronicity. This consideration would be more representative of secondary signs of MGD from the use of topical glaucoma medications that are typically 0.005% to 0.02% in the clinic [59]. 

This study provides novel insight into the distribution, density, and phenotype of IC populations that reside around MGOs. These MOIC populations were found to mostly express CD11c, with a smaller representation of MHC-II^+^ and Cx3cr1^+^ cells. The density of CD11c^+^ MOICs was greater in younger mice, but whether further changes in distribution occur in much older ages still warrants investigation. The number and morphology of ICs around the MGO niche were also found to be affected by a pro-inflammatory topical stimulus. Given the inflammatory nature of MGD and DED, these novel findings contribute to advancing scientific understanding of the meibomian gland functional unit, and how it is perturbed during ocular surface inflammation. Further investigations in this area may provide future insights into the processes that underlie these conditions.

## 4. Materials and Methods

### 4.1. Ethics and Animals

Animal ethics approval was obtained from the Animal Ethics Committee at the Florey Institute of Neuroscience and Mental Health, Melbourne, Victoria, Australia (18-093-UM and 21-032-UM). Healthy transgenic CD11c^eYFP^ mice were used (three-month-old, *n* = 7: 3 male and 4 female; seven-month-old, *n* = 8: 3 male and 5 female). CD11c is expressed by DCs in mice. Using CD11c^eYFP^ reporter mice, which express enhanced yellow fluorescent protein (eYFP) under the control of the CD11c integrin alpha X (Itgax) promoter [60], the presence and distribution of resident ICs could be clearly visualised along the eyelid margin using confocal microscopy. Cx3cr1^gfp/gfp^ mice (Cx3cr1-deficient) (three-month-old, *n* = 12: 7 male and 5 female; seven-month-old, *n* = 10: 3 male and 7 female) were also used. Cx3cr1 is a chemokine receptor expressed by subsets of resident ICs at mucosal sites [61,62,63]. Cx3cr1^gfp/gfp^ mice express enhanced green fluorescent protein (eGFP) under control of the Cx3cr1 locus, and thus Cx3cr1^+^ cells can be visualised based on their expression of eGFP. When two copies of GFP are inserted into both alleles of the Cx3cr1 gene, Cx3cr1 receptor function is impaired [64], allowing interrogation of the function of Cx3cr1 in mucosal immunity. Previous studies have reported varying roles for Cx3cr1 in the homeostatic recruitment of MHC-II^+^ ICs to epithelial tissues, including in the cornea [17], olfactory epithelium [65], and extension of lamina propria DC dendrites across the intestinal epithelium [49]. To assess whether Cx3cr1 has a similar role in the eyelid margin, we compared the density of MHC-II^+^ MOICs in three-month-old CD11c^eYFP^ and Cx3cr1^gfp/gfp^ mice.

The two transgenic strains were used to visualise and quantify resident CD11c^eYFP+^ and Cx3cr1^gfp/gfp+^ cells along the eyelid margin. All animals were housed at the Florey Institute of Neuroscience and Mental Health Animal Facility, Victoria, Australia, in a pathogen-free environment at 50% relative humidity (RH) with an 8% RH dead band and constant temperature 22 °C, with a 12 h light-dark cycle.

### 4.2. Model of Benzalkonium Chloride (BAK)-Induced Inflammation

To investigate whether the presence of MOICs was influenced by local inflammation, corneas and eyelids from three-month old CD11c^eYFP^ mice were examined after seven days of daily topical application of the preservative BAK (Figure 5A–C), or a saline control (Figure 5E–G). Equal numbers of male and female CD11c^eYFP^ mice (aged three months, *n* = 6 per group) were used to assess the effects of benzalkonium chloride (BAK), a known inducer of corneal and conjunctival inflammation, on the density and distribution of MOICs. On day 0, mice were anaesthetised with ketamine (80 mg/kg)/xylazine (10 mg/kg), underwent OCT imaging, and then received a five-microlitre eye drop of either saline or 0.1% BAK to both eyes. On subsequent days, conscious mice received a five-microlitre eye drop of either saline or 0.1% BAK to both eyes once daily, for seven days. 

### 4.3. Sample Size

The characterisation of MOICs in mice has not been previously described in the literature. Sample size estimates for the present study were based on previously published studies in our laboratory investigating similar IC populations in the mouse corneal epithelium, where a sample size of 6–8 mice per group was sufficient to detect differences in healthy and injured corneas [27].

### 4.4. Wholemount Immunofluorescence

Mice were euthanised using an intraperitoneal injection of sodium pentobarbital (100 mg/kg). Eyelids were carefully dissected at the nasal and medial canthus and along the forniceal conjunctiva. To aid in post-collection orientation of the eyelid, an incision was made in the nasal region, closest to the presumed puncta, to serve as a geographic landmark during imaging. Eyelids were fixed in Zamboni’s fixative containing 1.6% paraformaldehyde, <1.5% picric acid, mono- and disodium buffer salts, pH 7.3, 0.1 M (Australian Biostain Pty Ltd., Victoria, Australia) overnight at 4 °C, and then washed in PBS. Eyelids from the right eye were incubated overnight at room temperature under constant agitation with primary monoclonal rat anti-MHC-II antibody (1:200, #336999, BD Biosciences, Franklin Lakes, NJ, USA) in PBS + 5% goat serum + 0.5% Triton X-100. After PBS wash (3 × 5 min), right eyelids were incubated with secondary antibodies goat anti-rat Alexa Fluor 647 (A-21247, 1:500, Invitrogen, Waltham, MA, USA) and both left and right eyelids with Alexa Fluor conjugated phalloidin 568 (1:100, A12380, BD Biosciences, Franklin Lakes, NJ, USA) and Hoechst (1:500, Sigma, St. Louis, MO, USA) at room temperature for 90 min on an agitator. Immunostained eyelids were cover-slipped using aqueous mounting medium, with the palpebral conjunctiva and eyelid margin facing anteriorly.

For the BAK model, corneas from the right eye were dissected and fixed in 4% paraformaldehyde overnight at 4 °C and then washed in phosphate-buffered saline (PBS) (3 × 5 min) and incubated in Hoechst (1:500; Sigma-Aldrich, St. Louis, MO, USA) in PBS for 90 min at room temperature. CD11c-eYFP expression was used to visualise and analyse DC density and morphology in the corneal epithelium.

### 4.5. Spectral Domain Optical Coherence Tomography (SD-OCT)

SD-OCT imaging (Bioptigen Envisu R22200 VHR; Bioptigen, Inc., Durham, NC, USA, with the rodent alignment stage (AIM-RAS)) was used to measure central corneal thickness (CCT) immediately after euthanasia at day seven. Rectangular scans (3 mm × 3 mm; 1000 A-scans/200 B-scans) were captured with an 18 mm telecentric lens. Corneal epithelial and total thickness in the central cornea (CCT) were quantified using ImageJ software (http://imagej.nih.gov/ij/); (National Institutes of Health, Bethesda, MD, USA) by measuring the distance from the tear film to the anterior border of the stroma (epithelial thickness) and the endothelium (CCT).

### 4.6. Image Collection and Analysis

Eyelid–conjunctival flatmounts were imaged using confocal microscopy (SP8, Leica Microsystems, Buffalo Grove, IL, USA). A 10 µm z-stack scan from the superficial epithelium of the MGO was acquired using a 40×, 1.30 NA oil objective lens. Given the asynchronous nature of MG activity across the eyelid [8], and the effect of ageing on MG immune status [44,45], we considered the potential influence of six anatomical eyelid regions (i.e., superior vs. inferior, which were then also stratified by eccentricity: nasal vs. central vs. temporal) on MOIC density (Figure 1A) (0.35 µm step size; 1024 × 1024 pixel resolution). Scans of two adjacent MGOs in each region were obtained with nasal corresponding to the first two MGOs, and the two most temporal MGOs of the eyelid were analysed in the temporal region. The central two MGOs were defined to begin at half the number of total MGOs of an individual eyelid, i.e., $\frac{\mathrm{total}\;\mathrm{no}.\mathrm{of}\;\mathrm{glands}}{2}$. If the total number of MGOs for an individual eyelid was an odd number, then the halfway point of the total number of MGOs minus one denoted the first central gland (i.e., $\frac{\mathrm{total}\;\mathrm{no}.\mathrm{of}\;\mathrm{glands}-1}{2}$) (Figure 1C). The first nasally located circular structure was assumed to be the lacrimal punctum and excluded from the analysis. The investigator was masked to the identities of eyelids in the BAK- and saline-exposed groups.

### 4.7. Corneal Imaging

To determine corneal CD11c^+^ DC density and morphology, flatmounts were imaged using fluorescence imaging (Leica Thunder Imaging systems with Computational Clearing, Buffalo Grove, IL, USA). A montage image of corneal quadrants was captured using a X20, 0.8 NA dry objective lens. The investigator was masked to treatment groups.

### 4.8. Image Analysis

As MGO area is non-uniform, IC counts were normalised to MGO area to derive a density (cells/mm^2^) value. The outer MGO perimeter was traced using the “Polygon Selections” tool in ImageJ software (http://imagej.nih.gov/ij/); (National Institutes of Health, Bethesda, MD, USA). The outermost cells of the MGO were determined by the F-actin signal (Figure 1D). The Feret radius (half Feret diameter) was added onto the circumference of the outer MGO perimeter to determine a peri-orifice area, as previously described [10]. MOICs within the peri-orifice area were manually counted using the cell counter plugin in ImageJ (Figure 1E). MOICs with dendrites extending into the peri-orifice area, and which had a visible cell body outside the denoted peri-orifice area, were considered as associating with the MGO and were included in the cell count. 

To visualise the morphological characteristics of the MOICs at different depths, false coloured depth projections were generated in ImageJ (Figure 1F and Figure 2A,I). The CD11c^+^ MOIC density was also determined in the superficial 5 µm vs. deeper 5 µm zones, i.e., 0–5 µm and 5–10 µm, by manually counting the number of MOICs in the first 15 frames (0–5 µm) and last 15 frames (5–10 µm) of the z-stack. CD11c^+^ MOIC field area was traced using the “Polygon Selections” tool in ImageJ by tracing the furthermost tips of each dendrite using similar methods as previously described [27]. Each dendrite was followed by scanning the depth of the z-scan. Up to five CD11c^+^ MOICs were measured for each MGO, by measuring each MOIC whose cell body appeared the most superior first. If multiple cell bodies were equally visible in a particular frame, the first five MOICs were selected for analysis using a clockwise approach, beginning at the top left side of the image.

Corneal CD11c^+^ DC density was determined by manually counting the number of DCs in the peripheral and central cornea using the cell counter plugin in ImageJ, and field area (mm^2^) measured using the “Polygon tool” in ImageJ. Corneal area was also measured using the “Polygon tool” with the total corneal area measured following the perimeter of the quadrant and just anterior to the limbus. The radius of the cornea was measured using the line tool from the apex of the cornea to the anterior limbus, with most quadrants at approximately 1.8 mm. Central corneal area was determined as an approximately 0.9 mm radial area from the corneal apex. Peripheral area was determined by subtracting the central area from the total area (Figure S4). To determine morphological differences, up to 10 DCs were measured in the peripheral and central corneas, as previously described [27,66]. Briefly, for the central cornea, DCs were selected beginning at the apex, or tip of the quadrant, and followed a “Z” pattern until up to 10 DCs or all DCs in this area were measured (whichever came first). For the peripheral cornea, DCs were selected beginning at the left-most peripheral edge, following the perimeter to the right until 10 DCs were measured.

### 4.9. Flow Cytometry

Eyelid margins from 12–14-week-old CD11c^eYFP^ and Cx3cr1^gfp/gfp^ mice were dissected free from the palpebral conjunctiva and placed into Dulbecco’s phosphate-buffered saline (DPBS, Sigma Aldrich, St. Louis, MO, USA) and kept on ice. Due to the low number of eyelid margin resident immune cells, four eyelids from *n* = 3 mice were pooled, as triplicates for CD11c^eYFP^ mice (total, *n* = 9 mice), and replicates for Cx3cr1^gfp/gfp^ mice (total, *n* = 6 mice). Tissues were mechanically disassociated using a pestle and single cell suspensions, passed through a 100 µm cell strainer and incubated in DPBS containing primary antibodies Cx3cr1-BV421 (Becton Dickinson, Z8-50, for CD11c^eYFP^ mice), CD11c-BV421 (BD, HL3, for Cx3cr1^gfp/gfp^ mice), CD45-PE (Biolegend, 30-F11), and MHC-II APC (eBioscience, M5/114.15.2) on ice for 20 min. Single cell suspensions of spleen cells were included for compensation and unstained controls. Cells were then washed twice with PBS, resuspended in PB, and run on a CytoFlex LX flow cytometer (Beckman Coulter, Indianapolis, IN, USA). Low-angle and orthogonal light scatter were used to exclude dead cells and debris, forward scatter height (FSC-H) vs. forward scatter area (FSC-A) was used to exclude doublets, CD45 positivity was used to exclude non-immune cells, and electronic compensation was utilised to correct for spectral overlap between FITC/PE. Data were analysed using FlowJo 10.8.1 for Windows (Becton Dickson, Mountain View, CA, USA). Due to the low cell numbers and the need to pool multiple eyelids, statistical comparisons were not performed. Data are presented as triplicates representing three pools of 12 eyelids (CD11c^eYFP^) and two pools of 12 eyelids (Cx3cr1^gfp/gfp^ mice). Both males and female mice were used.

### 4.10. Statistical Analysis

GraphPad Prism (version 8.0; GraphPad Software, Inc., La Jolla, CA, USA) software was used to perform the statistical analyses. The mean MOIC density of the two adjacent MGOs in each region was calculated and compared with the other regions of the eyelid within each age group. Age comparisons (three-month-old versus seven-month-old) were also considered in relation to eyelid region (superior versus inferior). When comparing superior and inferior eyelid parameters among mice in the same age group, a two-way ANOVA was performed, matching by region and location of the eyelid (superior or inferior). When comparing data between different age groups, the superior and inferior eyelid MOIC densities were pooled and analyses were compared in relation to eyelid region only (nasal versus central versus temporal) using a two-way ANOVA, matching by region (nasal, central, and temporal). As region and location are matched data from the same mouse, sphericity was assumed. To determine the source of inter-factor difference, Šidák’s multiple comparisons test was used. 

Data normality was tested using the Shapiro–Wilk test for the following data sets and appropriate analyses described below. For three-month-old overall region eccentricity, after pooling superior and inferior eyelid data, CD11c^+^ MOIC density passed normality and a one-way ANOVA was used. For overall seven-month-old CD11c^+^ MOIC density and three and seven-month-old Cx3cr1^+^ MOIC density, data were non-parametric and a Friedman test was used for analyses. When comparing IC densities in the upper 5 µm to the lower 5 µm of the duct, a paired *t*-test was performed, as data were parametric. A Mann–Whitney U test was performed for CD11c^+^ MOIC field area, CD11c^+^ DC field area, and total CCT comparisons between BAK and saline controls as data were non-parametric. These data are presented as median [inter-quartile range]. Unpaired *t*-tests were performed for corneal epithelial thickness to total CCT ratio (%) and corneal CD11c^+^ DC density comparisons between BAK and saline controls.

All data are presented as mean ± standard deviation (SD) unless otherwise stated. Statistical significance was defined by *p* < 0.05 and is indicated in graphs by asterisks (* *p* ≤ 0.05; ** *p* ≤ 0.01; *** *p* ≤ 0.001; **** *p* ≤ 0.0001).

## Data Availability

The data sets generated during and/or analysed during the current study are available from the corresponding author on reasonable request.

## Supplementary Materials

The following supporting information can be downloaded at: https://www.mdpi.com/article/10.3390/ijms23179589/s1.
