# Supplementary material for: Topographical Distribution and Phenotype of Resident Meibomian Gland Orifice Immune Cells (MOICs) in Mice and the Effects of Topical Benzalkonium Chloride (BAK)
Source: Int J Mol Sci. 2022 Aug 24;23(17):9589. doi: 10.3390/ijms23179589 (PMC9455816; doi:10.3390/ijms23179589)
Supplement: Supplementary file 1 [file ijms-23-09589-s001.zip › ijms-1791840-supplementary.pdf]

Supplementary Material

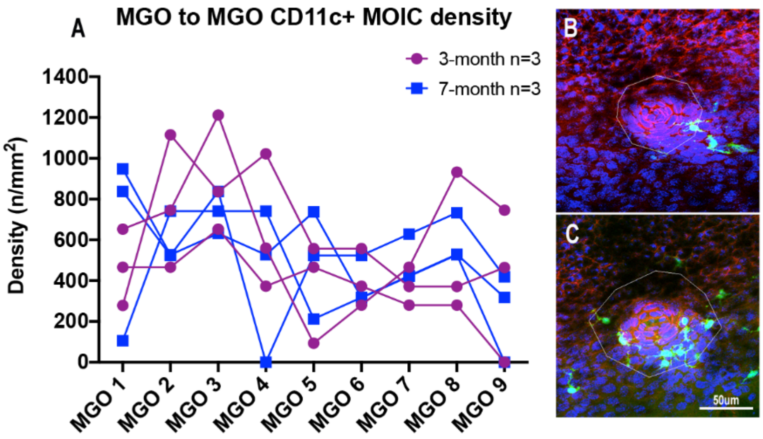

Figure S1. Individual variability between each MGO. (A) Graphic representation of $CD11c^+$ MOIC density (mean ± SD) along the eyelid margin (i.e., from one MGO to the next MGO, excluding inter-gland space) in individual three-month and seven-month old mice (n=3 per group). (B) and (C) Representative images of two immediately adjacent MGOs with large variability in $CD11c^+$ MOIC density. Scale bar applies to both confocal images.

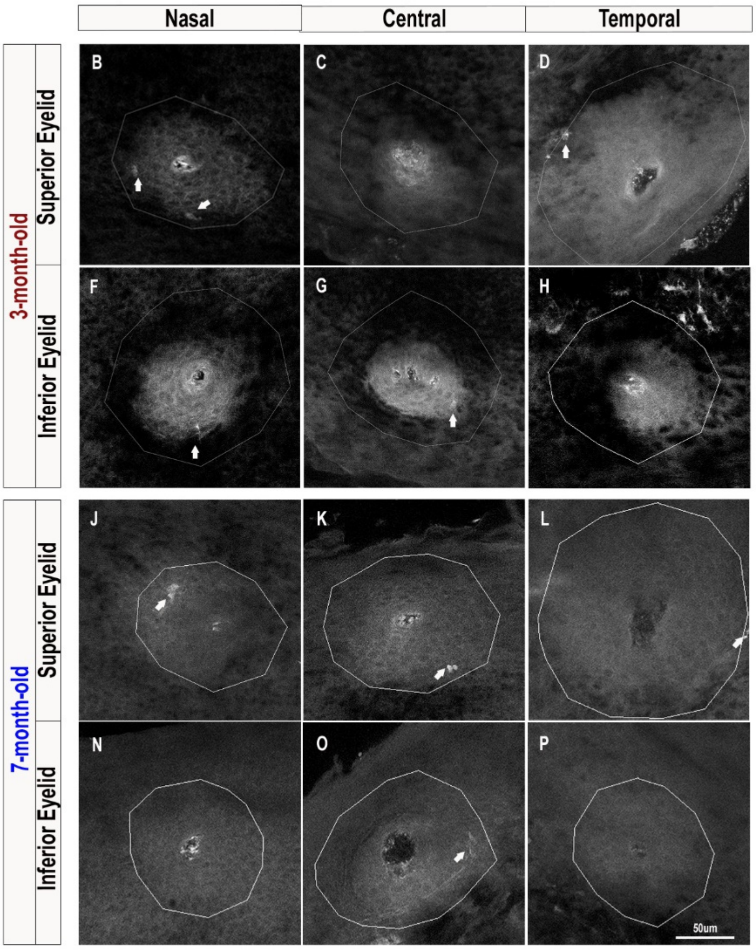

Figure S2. Confocal images of Cx3cr1⁺ IC in the 647-channel, corresponding to images in Figure 3. Scale bar in (H) applies to all images.

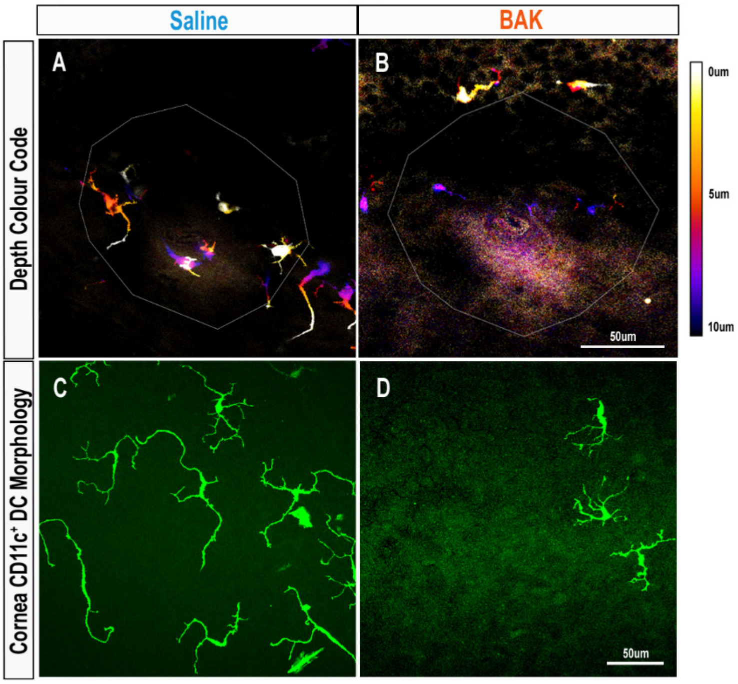

Figure S3. Depth colour coded images of MGO in BAK-treated (A) CD11c^{eYFP} mice and saline-treated controls (B). Scale bar in (B) also applies to (A). Representative confocal images of peripheral corneal CD11c⁺ DCs in saline control (C) vs BAK (D). Scale bar in (D) also applies to (C).

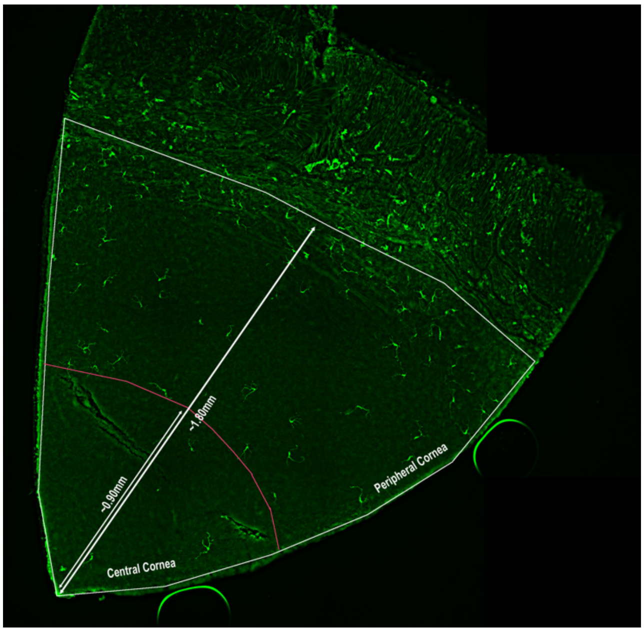

Figure S4. Diagram detailing corneal areas (central and peripheral). The radius of the cornea is measured approximately 1.80mm, with the central cornea defined as approximately 0.90mm radial area.

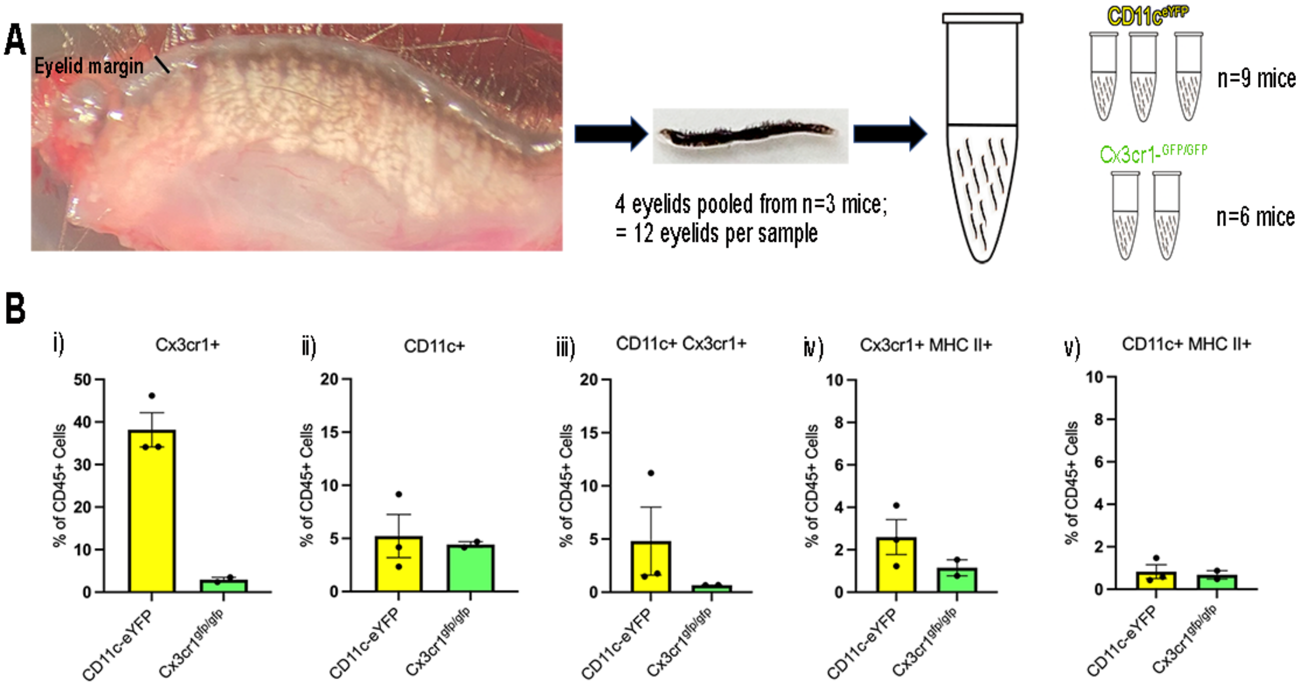

Figure S5. Flow cytometry of mouse eyelids. (A) Flow diagram depicting flow cytometry methodology. (B) Graphical representation of the percentage of $CD45^{+}$ immune cells and associated phenotypes of cells isolated from eyelid margins (Cx3cr1, CD11c, MHC-II). Each dot point on the graphs represents 12 pooled eyelids from n=3 mice.
